# Vanadium(V) oxide clusters synthesized by sublimation from bulk under fully inert conditions†
†Electronic supplementary information (ESI) available: Details of the mass spectrum, nozzle temperature effects on droplet size distribution, additional DFT results for the charged (V_2_O_5_)^+^*n* structures, minimum energy geometries in Cartesian coordinates. See DOI: 10.1039/c8sc05699d


**DOI:** 10.1039/c8sc05699d

**Published:** 2019-02-25

**Authors:** Maximilian Lasserus, Martin Schnedlitz, Roman Messner, Florian Lackner, Wolfgang E. Ernst, Andreas W. Hauser

**Affiliations:** a Institute of Experimental Physics , Graz University of Technology , Petersgasse 16 , A-8010 Graz , Austria . Email: andreas.w.hauser@gmail.com ; Email: wolfgang.ernst@tugraz.at ; Fax: +43 316 873 108140 ; Tel: +43 316 873 8157 ; Tel: +43 316 873 8140

## Abstract

Oxide nanoparticles in the size range of a few nanometers are typically synthesized in solution or *via* laser ablation techniques, which open numerous channels for structural change *via* chemical reactions or fragmentation processes. In this work, neutral vanadium oxide nanoparticles are instead synthesized by sublimation from bulk in combination with a pickup by superfluid helium droplets. Mass spectroscopy measurements clearly demonstrate the preservation of the bulk stoichiometric ratio of vanadium to oxygen in He-grown nanoparticles, indicating a tendency towards tetrahedral coordination of the vanadium centers in finite geometries. This unexpected finding opens up new possibilities for a combined on-the-fly synthesis of nanoparticles consisting of metal and metal-oxide layers. In comparison to mass spectra obtained *via* direct ionization of vanadium oxide in an effusive beam, where strong fragmentation occurred, we observe a clear preference for (V_2_O_5_)_*n*_ oligomers with even *n* inside the He nanodroplets, which is further investigated and explained using the electronic structure theory.

## Abstract 2

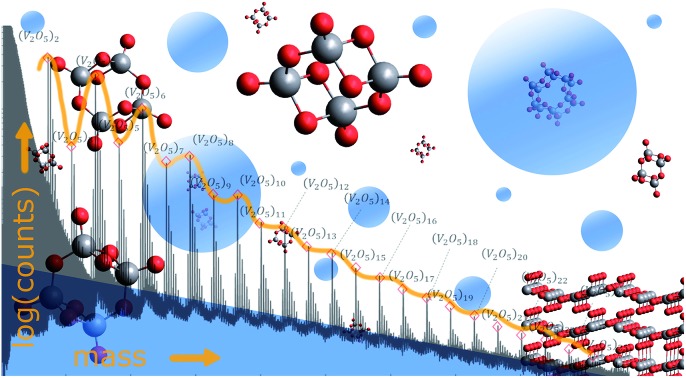
While laser ablation in combination with electron impact mass spectroscopy yield numerous fragments and reaction products, helium-mediated mass analysis reveals the sublimation from bulk in units of (V_2_O_5_)_2_.

## 1 Introduction

Vanadium oxides, a prominent class of the early 3d transition metal compounds, have been objects of great interest in the past few decades due to their very broad range of oxidation states. This feature, together with the comparatively high abundance of vanadium, makes this group of oxides highly valuable for catalysis applications such as NO_*x*_ reduction by NH_3_ (ref. 1) or the oxidation of hydrocarbons,^2^,^3^ and suggests new possibilities for the design of optical switching devices, waveguides,^1^,^4^ and cathodes for Li-ion battery cells.^5^

This diversity of possible oxidation states also implies a large variety of geometric and electronic structures. Following the discovery of vanadium oxide nanotubes in 1998,^6^,^7^ this particularly broad field of research has been extended by yet another facet: On the nanoscale, effects such as structural finiteness, large surface to volume ratio, and strong substrate or solvent interactions add substantially to the complexity of these materials. From the perspective of future applications, the fabrication of vanadium oxides in a nanostructured form is a highly attractive objective. Crucial for the development of such nanomaterials at an industrial level is a better understanding of the underlying formation processes, which has triggered several review articles on the subject, focusing on techniques such as physical vapor deposition^8^ or the hydrothermal treatment of aqueous solutions.^9^ In general, oxide nanoparticles are typically synthesized in solution, *e.g. via* sol–gel techniques, microemulsions, micelle/reverse micelle methods or precipitation.^10^–^18^ For the production of smallest clusters, laser vaporization is often the method of choice.^19^ In order to allow studies in the condensed phase, it may be combined with a flow reactor setup.^20^

A problem common to all these approaches is the strong impact of the chosen technique on the structural outcome in the nanometer range. In the case of synthesis in solution, the nanoparticles are typically coated by ligands, which not only affects the final geometries and actual properties but also limits their usability for follow-up-treatments of other materials to some extent, in particular if high purity is desired. Laser vaporization, on the other hand, comes at the cost of undesired byproducts and often highly reactive fragments. In the case of vanadium oxide, given its high flexibility of possible oxidation states, this implies the simultaneous synthesis of a large variety of stable neutral and charged clusters with various stoichiometries.^21^–^32^ Currently, numerous efforts are made to understand how certain gas phase stoichiometries translate into the desired ratios in the liquid for follow-up coating processes.^20^

An alternative synthesis is presented in this article. It can be considered as an offspring of helium nanodroplet isolation spectroscopy,^33^–^35^ where helium nanodroplets (He_N_) are extensively utilized as superfluid, inert containers for the spectroscopy of atoms, molecules and small clusters. Typically, this is achieved *via* a pickup of the target molecules by a beam of helium droplets, created in the process of a supersonic expansion of helium through a cooled nozzle. Due to the extremely low helium cluster temperature of 0.37 K (ref. 36) and the practically inert environment provided by the droplets, it is even possible to synthesize very weakly bound or highly reactive species, which can then be studied on-the-fly *via* optical methods such as laser-induced fluorescence spectroscopy or mass spectrometry. Alternatively, the structures can also be deposited on substrates for follow-up studies *via* electron microscopy,^37^–^39^ electron energy loss spectroscopy,^40^ energy-dispersive X-ray spectroscopy^41^ or surface diffraction methods.^42^

In this article, we apply the helium droplet technique to the problematic but highly interesting case of vanadium oxide. We will show that a pickup of pure, neutral vanadium(V) oxide particles from vapour over the heated bulk material is possible, and demonstrate that a follow-up ionization of these particles inside the He droplets is less destructive than direct ionization in an effusive beam. This is highly advantageous, as it allows us to distinguish fragments, undesired byproducts of the ionization process, from the actual particle distribution of vanadium oxide vapour over heated bulk, and helps to clarify previous discussions on stability and abundance of certain oligomers. The correct stoichiometry for all oligomers (V_2_O_5_)_*n*_ for *n* up to 25 is confirmed *via* time-of-flight (TOF) mass spectra, which is a particularly important result for the ongoing research on the synthesis of mixed-metallic core–shell nanoparticles with the same technique,^37^,^43^,^44^ as it opens up the possibility of an additional coating with metal-oxide catalysts in future experiments.

## 2 Results and discussion

**Fig. 1:**
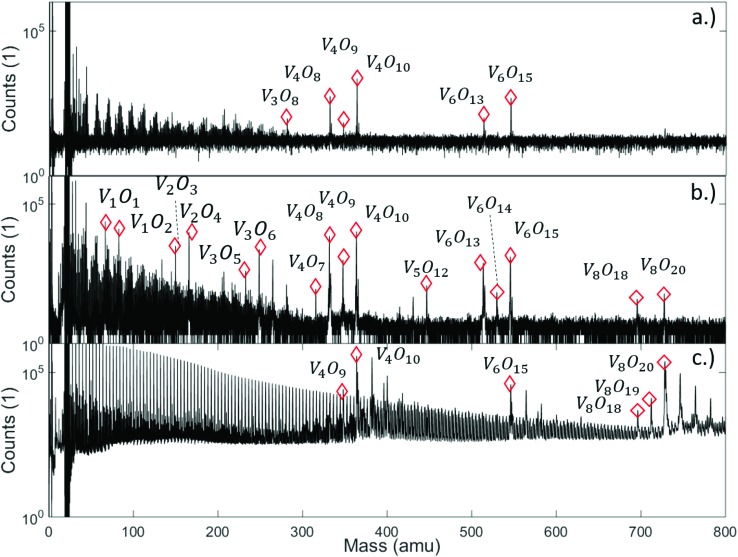
Mass spectra of vanadium oxide sublimated from V_2_O_5_ powder, and ionized under various conditions: (a) effusive source, direct ionization *via* electron impact with 20 eV, (b) effusive source, direct ionization *via* electron impact with 89 eV, and (c) *via* helium droplet beam (stagnation pressure 20 bar, nozzle temperature 9.2 K), indirect ionization of the He-immersed particles at 89 eV. Destructive effects of the electron beam at higher voltage are clearly visible (b), leading to numerous fragments scattered around the peaks of the (V_2_O_5_)_*n*_ series, while the helium-droplet-mediated ionization (c) appears to be the least invasive method of detection. It also shows a regular sequence with a spacing of 4 amu in the left half of the spectrum due to the He_N_ cluster fragmentation, and adsorption peaks of H_2_O are visible after each peak of the (V_2_O_5_)_*n*_ series. Note the lack of a pronounced peak at the mass of V_2_O_5_ in all spectra. A full description of the spectra with labels for all peaks can be found in Fig. S3† of the ESI.

We begin our discussion with a direct comparison of three time-of-flight mass spectra obtained in different experimental scenarios as presented in Fig. 1. The first graph, Fig. 1a, is obtained after direct ionization of an effusive vanadium oxide beam by electron impact at 20 eV. Two groups of peaks appear, which can be assigned to the structures (V_2_O_5_)_2_, (V_2_O_5_)_3_, and to fragments of both, with one or two oxygen atoms removed. In Fig. 1b, the same effusive beam is ionized by electron impact with an energy of 89 eV, an energy commonly applied in commercial mass spectrometers. It shows a similar mass distribution in the range between about 300 and 600 amu, but also the onset of the next multiple of the V_2_O_5_ unit, in this case (V_2_O_5_)_4_. From now on, we will refer to the clusters built from this unit as (V_2_O_5_)_*n*_ oligomers. In contrast, the spectra below 364 amu look entirely different for both choices of electron impact voltage. The appearance of numerous fragments for ionization at 89 eV, while none of these peaks is visible at 20 eV, indicates that direct ionization at larger energies is a highly invasive method of detection, altering the actual distribution of cluster sizes significantly. However, the spectrum obtained by indirect ionization within the helium droplets, as illustrated in Fig. 1c, suggests an even less destructive effect despite using the same electron impact voltage of 89 eV, as it shows very regular and nicely grouped patterns of peaks which can be assigned to oligomers with *n* = 2, 3 and 4. This experimentally well-documented^45^–^47^ and beneficial side-effect of He-droplet immersion is still not fully understood. For a tentative explanation, we refer to a recent review article^47^ on the matter, stating that the chances for a He-immersed molecule to survive ionization without fragmentation increase with droplet size, most likely due to a more efficient and rapid cooling of the vibrationally hot molecular ions.

**Fig. 2:**
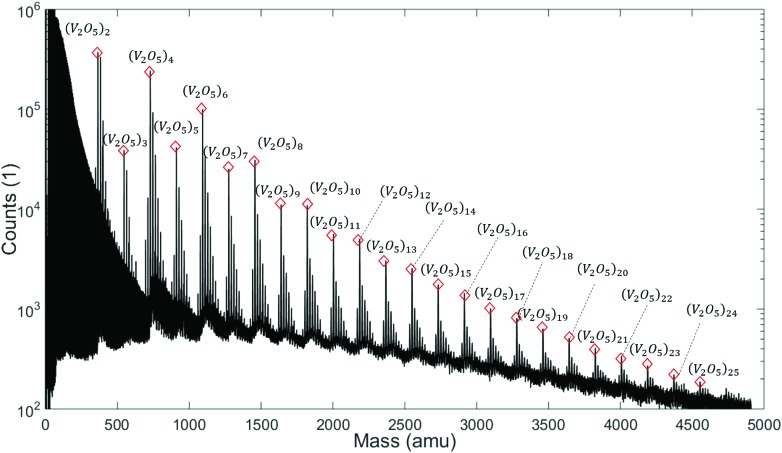
Mass spectra measured at a stagnation pressure of 20 bar and a nozzle temperature of 9.2 K. Peaks assigned to (V_2_O_5_)_*n*_ oligomers are marked with red diamonds. Additional peaks appear due to adsorption of water molecules. An oscillation between even and odd *n* is visible.

Note the lack of a clearly distinguishable V_2_O_5_ signal in all spectra shown in Fig. 1. This interesting detail will become highly relevant for the interpretation of the extended mass spectra presented in Fig. 2, which continues the He-droplet-mediated spectra towards higher masses up to 5000 amu. On the left side of the spectrum in Fig. 2 the helium droplet distribution is visible again, with peaks separated by exactly 4 amu (see Fig. S1† of the ESI for details), but in the relevant region above 330 amu the clearly distinct series of (V_2_O_5_)_*n*_ oligomer peaks dominates the spectra. Each of these main peaks is followed by a characteristic series of smaller signals with a mass difference of 18 amu. They stem from the inevitable pickup of water molecules as adsorbents, a typical contaminant in He droplet experiments.^48^ During heating, the desorption of molecular oxygen from bulk V_2_O_5_ can also be observed,^49^ giving rise to a higher background pressure of O_2_ (peak at 32 amu), and, as a direct consequence, the occurrence of molecular oxygen as a second yet barely noticeable contaminant of the V_2_O_5_ oligomers (see Fig. S1† of the ESI).

Two very interesting features become evident on the larger scale: First, the smallest oligomer is the (V_2_O_5_)_2_ dimer, which also exhibits the highest abundance. Second, the oligomers themselves show a preference for even numbers of *n*. Mass peaks of oligomers with even *n* are significantly higher than those for odd *n*. This oscillating pattern is very pronounced for smaller oligomers but gets weaker at higher masses. However, a difference of one to two orders of magnitude can be observed even up to *n* = 8. The first observation is in good agreement with previous studies on the evaporation of V_2_O_5_.^50^ The observed even–odd oscillations in *n* will be explained later in the text.

Two explanations are conceivable for the experimental results. The first is to assume the existence of magic numbers for the oligomers due to electronic or structural advantages in finite geometries, a well-known phenomenon which is typical and well documented *e.g.* for clusters formed by rare gas atoms (for geometrical reasons) or metal atoms (for electronic reasons). This hypothesis is easily tested using density functional theory (DFT) calculations on the free (V_2_O_5_)_2_ oligomers in the gas phase. Previous studies on neutral clusters,^51^ negatively^52^–^55^ or positively charged fragments,^32^,^55^ coordinated clusters or clusters in solution^20^ indicate a larger number of more or less stable stoichiometries, mostly with slight deviations from the stoichiometry found in the bulk material. In particular, the Sauer group provided a deep and systematic structural and vibrational analysis of vanadium oxide cluster cations to support gas phase infrared spectroscopy measurements.^56^ In this context, peroxo and superoxo species also were of special interest due to their role in re-oxidation processes during catalytic reactions.^57^ However, to the knowledge of the authors, no study has set a special focus yet on the formation process and the resulting size distribution of pristine vanadium oxide nanostructures with exact bulk stoichiometry. In helium droplet experiments, we find this stoichiometry as result of a chemically unperturbed phase transition from the solid to the gas phase under fully inert conditions. Closest to our current interest is a theoretical study of Vyboishchikov and Sauer,^51^ which compares the bulk structure and the neutral (V_2_O_5_)_*n*_ oligomers up to *n* = 12, but is not concerned with sublimation energies.

**Fig. 3:**
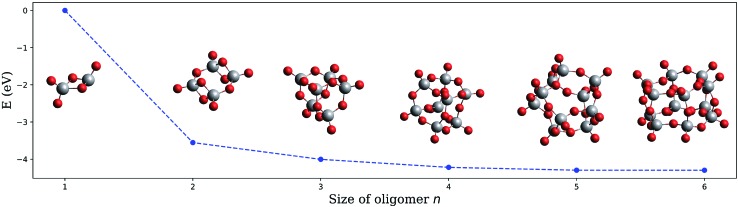
Electronic energy of the neutral (V_2_O_5_)_*n*_ clusters per building unit, plotted as a function of the oligomer size *n*.

The structures obtained in fully unconstrained energy minimizations with the *ω*B97X-V functional^58^ and their corresponding energies for oligomers with *n* = 1 to 6 are presented in Fig. 3, where we have divided the total electronic energy obtained for each oligomer by its corresponding number of building units. Structural comparison identifies ring closure of (V_2_O_5_)_*n*_ chains as the underlying formation principle. The neutral structures are very similar to those reported earlier except for (V_2_O_5_)_2_, where a ring-like structure, originally termed ‘4-square D_2h_’, is also slightly preferred over the tetragonal, Jahn-Teller-distorted *D*_2d_ arrangement named ‘4-tetra’.^51^,^54^ However, the curve in Fig. 3, binding energy per unit as a function of *n*, monotonically decreases, which refutes the assumption of magic numbers in neutral cluster formation. This test has been repeated for the cations with a similar outcome (see Fig. S4† in the ESI for details), which further excludes a varying stability of the oligomers after ionization as a valid argument.

This leaves us with the second hypothesis only, which assumes a direct impact of the actual sublimation process on the size distribution of the free gas oligomers. Vanadium(V) oxide crystallizes in an orthorhombic layer-type structure (*Pmmm*). In the first attempt to study the solid to gas phase transition we performed periodic DFT calculations on a pristine single layer of bulk vanadium(V) oxide with the PBE functional. Next, we performed a stepwise removal of V_2_O_5_ building units and repeated the optimization of the layer in order to obtain reconstruction energies after sublimation. Together with the energy calculations for the fully relaxed free gas oligomers, with the same computational approach we can estimate the electronic energies for the sublimation of *n* units *via*

**Fig. 4:**
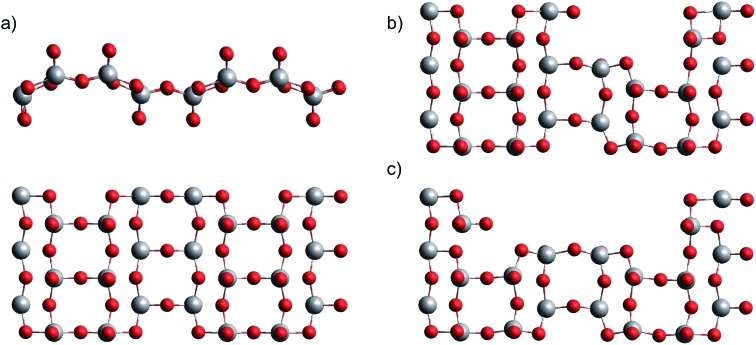
Single layer of bulk vanadium(V) oxide in two views (a), after removal of one (b) and two (c) V_2_O_5_ units.

1 *E*(*n*) = (*E*_surf–*n*u_ + *En*cluster) – *E*_surf_,

where *E*_surf_ and *E*_surf–*n*u_ are the total electronic energy of the surface before and after the removal of *n* units, respectively, and *En*cluster denotes the electronic energy of a free gas cluster built from *n* units. Structures of the pristine as well as the distorted layers are depicted in Fig. 4. Our choice of which V_2_O_5_ unit to remove follows the minimum energy criterion. To keep the inevitable bias, introduced by a supercell of finite size, at a minimum, we limit our study to a removal of not more than four units from the bulk surface. In the next step, these energy differences are corrected for thermochemistry. Assuming the same entropy contributions for the pristine and the distorted bulk surface layer, such a correction reduces to the evaluation of entropy and enthalpy contributions for the free gas oligomers. For higher accuracy, we use the values obtained *via* frequency calculations obtained with the *ω*B97X-V functional,^58^ calculated at *T* = 1000–1400 K and a pressure of 10^–3^ mbar, and add them to the electronic energies obtained in the periodic calculations. Entropy values have been corrected according to ref. 59 for improved estimates of contributions from low-lying vibrational frequencies.

**Fig. 5:**
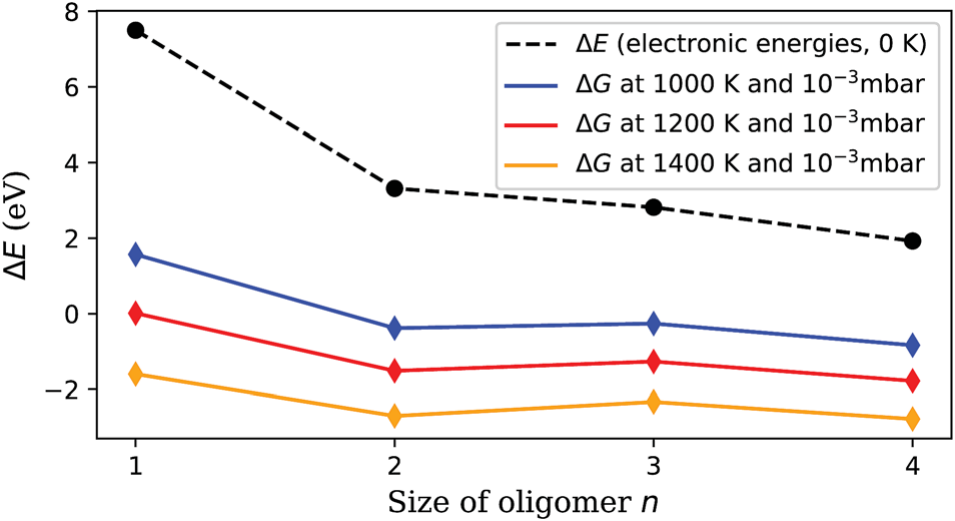
Sublimation energy per V_2_O_5_ unit, calculated at zero K and under experimental conditions. Sublimation starts around 1000 K, but is still not feasible for single units at this temperature. (V_2_O_5_)_2_ detachment becomes preferred at higher temperatures, which explains the much higher abundance of (V_2_O_5_)_*n*_ clusters with even *n* in Fig. 2.

The resulting Gibbs energy differences are plotted in Fig. 5, together with the electronic energy differences at 0 K, and scaled to energies per detached V_2_O_5_ unit. We find a clear preference for the sublimation of pairs over the detachment of single units. With increasing temperature, the sublimation of dimers becomes most feasible (followed by tetramers and trimers), which immediately explains the abundance of the (V_2_O_5_)_2_ dimer in the spectrum. Obviously, forming dimers upon sublimation is a compromise between minimizing the reconfiguration costs of the detachment and maximizing the entropy by releasing as many particles as possible. The lower cost for the sublimation of pairs overcompensates the bisection of the number of free gas particles.

With this information the last piece of the puzzle is placed easily: the preference for even oligomers must be a direct consequence of this high abundance of dimers. The pickup probability can be estimated based on vapour pressure, average size of the helium droplets, and the length of the pickup zone (15 mm). For the chosen conditions, the occasional pickup of several dimers by the same droplet is very likely. We therefore conclude that larger oligomers are formed by typical coagulation processes inside the helium droplets^60^ after a series of pickup events of mostly (V_2_O_5_)_2_ dimers. Further evidence for the growth of larger oligomers from these dimers *via* coagulation is given in the ESI,† where mass spectra obtained at different nozzle temperatures are compared. With decreasing nozzle temperature the He droplets increase in size and collect larger amounts of (V_2_O_5_)_2_ units, which in turn allows for the synthesis of larger vanadium oxide structures and therefore extends the characteristic pattern of peaks in the spectra towards higher masses.

## 3 Conclusions

In summary, we have demonstrated that pure vanadium(V) oxide oligomers with correct stoichiometry can be produced by sublimation and follow-up pickup by superfluid helium droplets. Ionization, a necessary step for follow-up mass spectroscopy, is proven to be less destructive to the sublimated particles if they are immersed in He nanodroplets. Although the charge hopping process during ionization inside the droplets should deliver about the same excess energy as direct 20 eV electron bombardment, fragmentation seems to play a much smaller role. In agreement with other groups, we attribute this observation to the high cooling rate of the surrounding helium. The smallest building unit for the vanadium oxide oligomers observed in the mass spectra is (V_2_O_5_)_2_, which is also the most abundant species in the helium droplet beam so far. We further observe a clear preference for (V_2_O_5_)_*n*_ oligomers with even *n* inside the He nanodroplets. This is explained by a computational study on a single layer of bulk vanadium(V) oxide, which suggests a preferred sublimation of (V_2_O_5_)_2_ dimers. These dimers serve as building blocks for the coagulation of larger structures inside the droplets. Experimentally, these novel findings indicate the emergence of a new method for the *in situ* coating of metallic nanoparticles with a metal oxide of high industrial relevance, as metal clusters can be synthesized simultaneously with the same experimental technique *via* a sequential pickup. Follow-up experiments on vanadium oxide-coated metal clusters are currently under way.

## 4 Methods

### Experimental setup

**Fig. 6:**
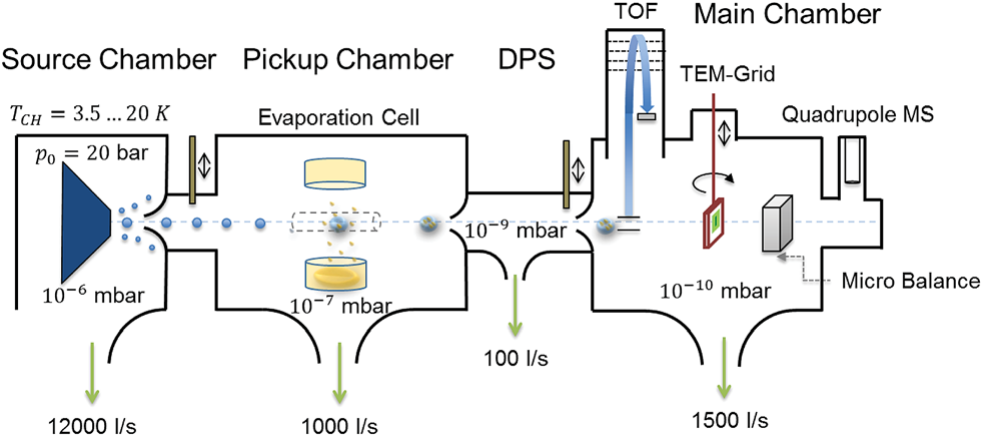
Schematic of the experimental setup used for the synthesis of (V_2_O_5_)_*n*_ clusters inside liquid He droplets; see the text for details.

Highly purified He gas (99.9999%, 20 bar) is expanded through a 5 μm nozzle into a high vacuum with a base pressure of ≈10^–5^ mbar. The nozzle temperature is set at around 9.2 K in order to produce helium droplets consisting of about 10^6^ He atoms, formed in the process of the supersonic expansion.^61^ The resulting droplet beam is cropped by a 400 μm skimmer before entering a separately pumped vacuum chamber for particle pickup. Details on the He droplet size distribution are given in Fig. S2 of the ESI.† A schematic of the whole apparatus is presented in Fig. 6. The setup consists of three vacuum chambers, one for the helium droplet production (source chamber), one for the pickup of vanadia fragments from the gas phase (pickup chamber) and one for cluster analysis (main chamber).

By resistive heating of V_2_O_5_ powder with a purity of 99.9%, kept in a small quartz crucible (Kurt Lesker EVC2Q), to temperatures between 1000 and 1200 K, a vapour pressure of ≈10^–3^ mbar is achieved.^62^ This determines, together with the He droplet size and the length of the cell, the probability of a particle pickup from the gas phase, which offers a convenient handle for the amount of material brought into the He droplet beam. Each pickup event causes the evaporation of helium atoms from the droplet. This attenuation of the helium beam is measured with a quadrupole mass spectrometer (Balzers QMA 200/QME 200). After particle pickup, the He beam is again collimated by a 2 mm skimmer before entering the measurement chamber at a base pressure of approximately 10^–10^ mbar. The beam crosses the ionization region of a time-of-flight mass spectrometer (Stefan Kaesdorf RFT 50) designed for measurements of heavy compounds. Ionization is achieved *via* electron impact, using electron energies of 89 eV, an average electron emission current of ≈10 μA and a repetition rate of 10 kHz. However, note that the short mean free path of electrons in liquid helium prevents direct ionization of impurities inside helium droplets consisting of about 10^5^ He atoms or more.^63^ Typically, the ionization of an impurity inside the droplet takes place *via* secondary processes such as charge hopping^64^ or Penning ionization,^47^ which impedes undesired fragmentation through electron impact. For measurements without the He beam we closed a valve between the source and pickup chamber, which leaves us with an effusive beam of vanadium oxide particles only. The detection chamber further contains a quartz crystal microbalance, which allows us to measure the total mass transported by the helium beam and acts as an additional analysis tool. For further details on the experimental setup we recommend reading of ref. 65.

### Computational details

Our computational approach for the free-gas oligomers is based on the combination of a less expensive method for pre-optimization and structural search, in our case the GFN-xTB method of Grimme,^66^ with the application of *ω*B97X-V,^58^ a range-separated hybrid GGA density functional with VV10 nonlocal correlation,^67^ as it is implemented in the Q-Chem program package.^68^ For O we use the polarized triple-zeta basis set of Weigend and Ahlrichs,^69^ for V the effective core potential and basis set of the LANL family.^70^

The vanadium(V) oxide surface is described *via* periodic DFT calculations, where the PBE functional^71^ is used in combination with projector-augmented-wave (PAW) pseudopotentials^72^,^73^ as implemented in the Quantum Espresso suite of programs.^74^ A supercell containing 12 V_2_O_5_ building units is chosen, with an initial geometry taken from ref. 75. The geometry is allowed to fully relax, and the box dimensions in the *x* and *z* directions are optimized. We obtain 22.741 and 10.710 Å, respectively. For the *y* or (010) direction a box length of 10 Å is chosen to decouple the minimally interacting layers in our attempt to simulate the bulk surface. Energy cutoffs (90 Ryd for the energy, 1080 Ryd for the density) and *y*-distances are tested in order to provide an accuracy of 0.05 eV or 1 kcal mol^–1^, which lies within the systematic error expected for the chosen functional.

## Conflicts of interest

There are no conflicts to declare.

## Supplementary Material

Supplementary information

## References

[cit1] Forzatti P. (2001). Appl. Catal., A.

[cit2] Mamedov E., Corberán V. C. (1995). Appl. Catal., A.

[cit3] Haber J., Witko M., Tokarz R. (1997). Appl. Catal., A.

[cit4] Chain E. E. (1991). Appl. Opt..

[cit5] Winter M., Besenhard J. O., Spahr M. E., Novák P. (1998). Adv. Mater..

[cit6] Spahr M. E., Bitterli P., Nesper R., Müller M., Krumeich F., Nissen H. U. (1998). Angew. Chem., Int. Ed..

[cit7] Nesper R., Muhr H.-J. (1998). Chimia.

[cit8] Schoiswohl J., Surnev S., Netzer F. P., Kresse G. (2006). J. Phys.: Condens. Matter.

[cit9] Livage J. (2010). Materials.

[cit10] Wang D., Xie T., Li Y. (2009). Nano Res..

[cit11] Gvishi R. (2009). J. Sol-Gel Sci. Technol..

[cit12] Kwon S. G., Hyeon T. (2008). Acc. Chem. Res..

[cit13] Rao C., Vivekchand S., Biswas K., Govindaraj A. (2007). Dalton Trans..

[cit14] Chaudret B., Philippot K. (2007). Oil Gas Sci. Technol..

[cit15] Lim J. K., Majetich S. A., Tilton R. D. (2009). Langmuir.

[cit16] Tang J., Redl F., Zhu Y., Siegrist T., Brus L. E., Steigerwald M. L. (2005). Nano Lett..

[cit17] Cushing B. L., Kolesnichenko V. L., O'Connor C. J. (2004). Chem. Rev..

[cit18] Rockenberger J., Scher E. C., Alivisatos A. P. (1999). J. Am. Chem. Soc..

[cit19] Dietz T. G., Duncan M. A., Powers D. E., Smalley R. E. (1981). J. Chem. Phys..

[cit20] Ard S., Dibble C., Akin S., Duncan M. (2011). J. Phys. Chem. C.

[cit21] Bergeron D. E., Castleman A. W., Jones N. O., Khanna S. N. (2004). Nano Lett..

[cit22] Moore N. A., Mitrić R., Justes D. R., Bonac[combining breve]ić-Koutecký V., Castleman A. (2006). J. Phys. Chem. B.

[cit23] Feyel S., Schröder D., Rozanska X., Sauer J., Schwarz H. (2006). Angew. Chem., Int. Ed..

[cit24] Molek K., Reed Z., Ricks A., Duncan M. (2007). J. Phys. Chem. A.

[cit25] Fielicke A., Rademann K. (2002). Phys. Chem. Chem. Phys..

[cit26] Wang L.-S., Wu H., Desai S. R., Lou L. (1996). Phys. Rev. B.

[cit27] Dong F., Heinbuch S., Xie Y., Rocca J. J., Bernstein E. R. (2009). J. Phys. Chem. A.

[cit28] Green S. M., Alex S., Fleischer N. L., Millam E. L., Marcy T. P., Leopold D. G. (2001). J. Chem. Phys..

[cit29] Pramann A., Koyasu K., Nakajima A., Kaya K. (2002). J. Phys. Chem. A.

[cit30] Yoder B. L., Maze J. T., Raghavachari K., Jarrold C. C. (2005). J. Chem. Phys..

[cit31] Fielicke A., Meijer G., Von Helden G. (2003). Eur. Phys. J. D.

[cit32] Asmis K. R., Meijer G., Brümmer M., Kaposta C., Santambrogio G., Wöste L., Sauer J. (2004). J. Chem. Phys..

[cit33] Toennies J. P., Vilesov A. F. (2004). Angew. Chem., Int. Ed..

[cit34] Callegari C. and Ernst W. E., in Handbook of High Resolution Spectroscopy, ed. F. Merkt and M. Quack, John Wiley & Sons, Chichester, 2011, pp. 1551–1594, 10.1002/9780470749593.hrs064.

[cit35] Tiggesbäumker J., Stienkemeier F. (2007). Phys. Chem. Chem. Phys..

[cit36] Hartmann M., Miller R., Toennies J., Vilesov A. (1995). Phys. Rev. Lett..

[cit37] Schnedlitz M., Lasserus M., Meyer R., Knez D., Hofer F., Ernst W. E., Hauser A. W. (2018). Chem. Mater..

[cit38] Schnedlitz M., Lasserus M., Knez D., Hauser A. W., Hofer F., Ernst W. E. (2017). Phys. Chem. Chem. Phys..

[cit39] Koh A. L., Bao K., Khan I., Smith W. E., Kothleitner G., Nordlander P., Maier S. A., McComb D. W. (2009). ACS Nano.

[cit40] Melzer M., Urban J., Sack-Kongehl H., Weiss K., Freund H.-J., Schlögl R. (2002). Catal. Lett..

[cit41] Slater T. J., Janssen A., Camargo P. H., Burke M. G., Zaluzec N. J., Haigh S. J. (2016). Ultramicroscopy.

[cit42] Schiffmann K. I., Fryda M., Goerigk G., Lauer R., Hinze P., Bulack A. (1999). Thin Solid Films.

[cit43] Lasserus M., Schnedlitz M., Knez D., Messner R., Schiffmann A., Lackner F., Hauser A. W., Hofer F., Ernst W. E. (2018). Nanoscale.

[cit44] Haberfehlner G., Thaler P., Knez D., Volk A., Hofer F., Ernst W. E., Kothleitner G. (2015). Nat. Commun..

[cit45] Lewis W. K., Applegate B. E., Sztáray J., Sztáray B., Baer T., Bemish R. J., Miller R. E. (2004). J. Am. Chem. Soc..

[cit46] Bartl P., Tanzer K., Mitterdorfer C., Karolczak S., Illenberger E., Denifl S., Scheier P. (2013). Rapid Commun. Mass Spectrom..

[cit47] Denifl S. (2013). Eur. Phys. J.: Spec. Top..

[cit48] Lindebner F., Kautsch A., Koch M., Ernst W. E. (2014). Int. J. Mass Spectrom..

[cit49] Milan E. F. (1928). J. Phys. Chem..

[cit50] Beattie I. R., Ogden J. S., Price D. D. (1978). Inorg. Chem..

[cit51] Vyboishchikov S. F., Sauer J. (2001). J. Phys. Chem. A.

[cit52] Bell R. C., Zemski K. A., Justes D. R., Castleman A. W. (2001). J. Chem. Phys..

[cit53] Feyel S., Schwarz H., Schröder D., Daniel C., Hartl H., Döbler J., Sauer J., Santambrogio G., Wöste L., Asmis K. R. (2007). ChemPhysChem.

[cit54] Santambrogio G., Brümmer M., Wöste L., Döbler J., Sierka M., Sauer J., Meijer G., Asmis K. R. (2008). Phys. Chem. Chem. Phys..

[cit55] Wu J. W. J., Moriyama R., Tahara H., Ohshimo K., Misaizu F. (2016). J. Phys. Chem. A.

[cit56] Asmis K. R., Sauer J. (2007). Mass Spectrom. Rev..

[cit57] Guimond S., Abu Haija M., Kaya S., Lu J., Weissenrieder J., Shaikhutdinov S., Kuhlenbeck H., Freund H.-J., Döbler J., Sauer J. (2006). Top. Catal..

[cit58] Mardirossian N., Head-Gordon M. (2014). Phys. Chem. Chem. Phys..

[cit59] Grimme S. (2012). Chem. – Eur. J..

[cit60] Hauser A. W., Volk A., Thaler P., Ernst W. E. (2015). Phys. Chem. Chem. Phys..

[cit61] Gomez L. F., Loginov E., Sliter R., Vilesov A. F. (2011). J. Chem. Phys..

[cit62] Farber M., Manuel Uy O., Srivastava R. (1972). J. Chem. Phys..

[cit63] Fursa D. V., Bray I. (1995). Phys. Rev. A.

[cit64] Ellis A. M., Yang S. (2007). Phys. Rev. A.

[cit65] Thaler P., Volk A., Knez D., Lackner F., Haberfehlner G., Steurer J., Schnedlitz M., Ernst W. E. (2015). J. Chem. Phys..

[cit66] Grimme S., Bannwarth C., Shushkov P. (2017). J. Chem. Theory Comput..

[cit67] Mardirossian N., Head-Gordon M. (2014). J. Chem. Phys..

[cit68] Shao Y. (2015). Mol. Phys..

[cit69] Weigend F., Ahlrichs R. (2005). Phys. Chem. Chem. Phys..

[cit70] Roy L. E., Hay P. J., Martin R. L. (2008). J. Chem. Theory Comput..

[cit71] Perdew J. P., Burke K., Ernzerhof M. (1996). Phys. Rev. Lett..

[cit72] Blöchl P. E. (1994). Phys. Rev. B.

[cit73] Kresse G., Joubert D. (1999). Phys. Rev. B.

[cit74] Giannozzi P., Baroni S., Bonini N., Calandra M., Car R., Cavazzoni C., Ceresoli D., Chiarotti G. L., Cococcioni M., Dabo I., Dal Corso A., de Gironcoli S., Fabris S., Fratesi G., Gebauer R., Gerstmann U., Gougoussis C., Kokalj A., Lazzeri M., Martin-Samos L., Marzari N., Mauri F., Mazzarello R., Paolini S., Pasquarello A., Paulatto L., Sbraccia C., Scandolo S., Sclauzero G., Seitsonen A. P., Smogunov A., Umari P., Wentzcovitch R. M. (2009). J. Phys.: Condens. Matter.

[cit75] Ketelaar J. A. A. (1936). Nature.

