# Supplementary material for: Vanadium(V) oxide clusters synthesized by sublimation from bulk under fully inert conditions
Source: Chem Sci. 2019 Feb 25;10(12):3473–80. doi: 10.1039/c8sc05699d (PMC6432649; doi:10.1039/c8sc05699d)
Supplement: Supplementary file 1 [file SC-010-C8SC05699D-s001.pdf]

Electronic Supplementary Material (ESI) for Chemical Science.
This journal is © The Royal Society of Chemistry 2019

Supporting Information: Vanadium(V) oxide clusters synthesized by sublimation from bulk at fully inert conditions

Maximilian Lasserus, Martin Schnedlitz, Roman Messner, Florian Lackner, Wolfgang E. Ernst,* and Andreas W. Hauser*

Institute of Experimental Physics, Graz University of Technology, Petersgasse 16, A-8010 Graz, Austria. Fax: +43 (316) 873-108140; Tel: +43 (316) 873-8157; +43 (316) 873-8140;

E-mail: wolfgang.ernst@tugraz.at; andreas.w.hauser@gmail.com

In the first section of this Supporting Information we present details of the mass spectrum shown in the main article, ranging from 320 amu to 400 amu. Section S2 discusses the change in the He droplet distribution due to different nozzle temperatures. Mass spectra with all peaks assigned to molecular structures are provided in Section S3. In Section S4, additional DFT results are presented for the charged $(V_2O_5)_n^+$ structures. Section S5 compares the ionization energies of the neutral clusters. The remaining sections S6 and S7 contain the minimum energy geometries in cartesian coordinates as obtained at the ωB97X-V (gas phase) or PBE (bulk) level of theory.

Contents

S1 Details of the mass spectra 3

S2 Droplet size distribution 4

S3 Fully assigned mass spectra 5

S4 Minimum energy comparison of the cationic species 5

S5 Ionization energies 6

S6 Geometries of the gas-phase species $(V_2O_5)_n$ 7

S6.1 Monomer . . . 7

S6.2 Dimer . . . 7

S6.3 Trimer . . . 8

S6.4 Tetramer . . . 9

S6.5 Pentamer . . . 10

S6.6 Hexamer . . . 11

S7 Bulk structures 14

S7.1 Unperturbed plane . . . 14

S7.2 One unit removed . . . 17

S7.3 Two units removed . . . 20

S1 Details of the mass spectra

In Figure S1 we show a detail of the mass spectra presented in Figure 2 of the main article, zooming into the area between 320 amu and 400 amu. Several peaks appear in close proximity to the signals assigned to the $(V_2O_5)_n$ clusters, which can be attributed to the adsorption of water molecules. For the $(V_2O_5)_2$ signal, an additional peak is visible which corresponds to a fragment lacking a single O atom. The regular sequence of unlabeled peaks with a spacing of 4 amu represents the He_N cluster distribution.

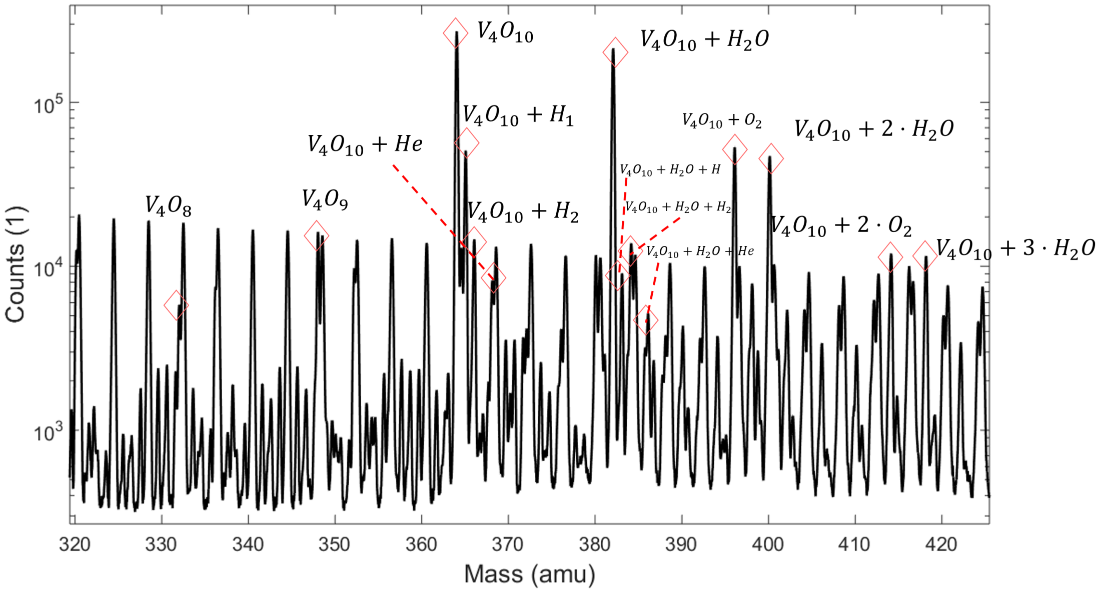

Figure S1: Magnified section of the mass spectra in Figure 2 of the main article. All peaks in direct neighborhood of the $(V_2O_5)_2$ signal have been assigned to various contaminants.

S2 Droplet size distribution

In the main manuscript the variation of the nozzle temperature was mentioned as a handle to control the He droplet size distribution. Figure S2 shows mass spectra as a function of the nozzle temperature. At lower nozzle temperatures the He droplets increase in size and are able to collect larger amounts of $(V_2O_5)_2$ units. This in turn enables the synthesis of larger vanadium oxide structures inside the droplets, and the characteristic pattern of $(V_2O_5)_n$ peaks in the mass spectra is continued towards higher masses. At 14 K only dimers are present, while larger oligomers start to appear at lower temperatures due to the increasing pickup cross section for the larger He droplets.

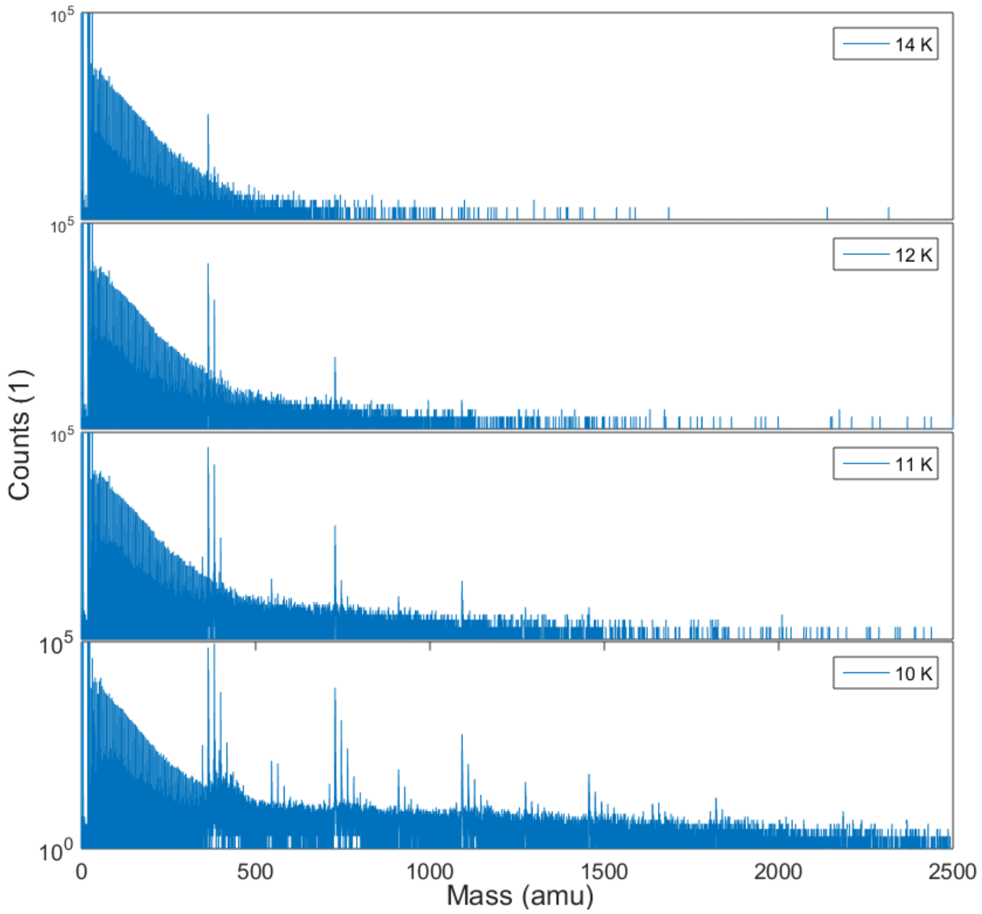

Figure S2: Mass spectra obtained for different nozzle temperatures. The temperature of the V_2O_5 crucible was kept constant during measurements.

S3 Fully assigned mass spectra

In Figure 1b of the main article several smaller peaks remained unassigned to avoid cluttering. A full assignment of the corresponding region is provided in Figure S3, where all peaks related to vanadium oxide structures are labelled and marked by red diamonds.

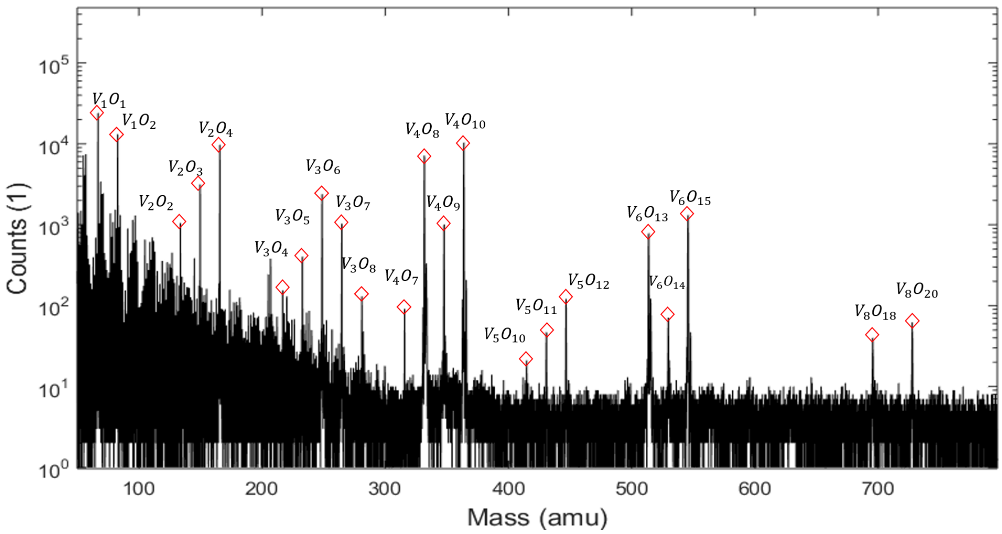

Figure S3: Same mass spectra as shown in Figure 1b of the main text for 89 eV, but with all peaks related to vanadium oxide marked by red diamonds.

S4 Minimum energy comparison of the cationic species

The same DFT procedure as described in the main article has been applied to the $(V_2O_5)_n^+$ cationic cluster species. In Figure S4 a monotonic increase of binding energy per unit can be observed, without any hint of oscillation with the number n of building units.

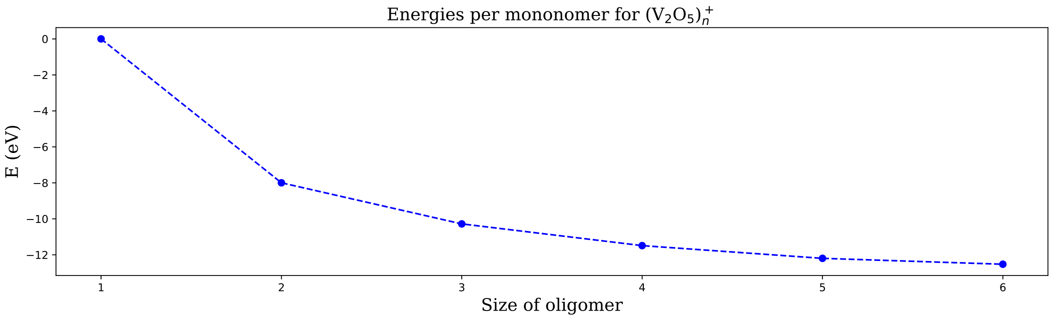

Figure S4: Electronic energy of cationic $(V_2O_5)_n^+$ clusters per building unit, plotted as a function of oligomer size n.

S5 Ionization energies

The ionization energies of the neutral clusters, obtained with the ωB97X-V functional, are plotted in Figure S5. Energies are calculated by taking the difference between electronic states containing $N-1$ and N electrons, evaluated at the minimum energy geometries of the neutral species. The stabilization of the electronic structure when going from the monomer to the dimer is significant. It is related to bond saturation and explains the large drop of binding energy per unit as shown in Figure 3 of the main manuscript.

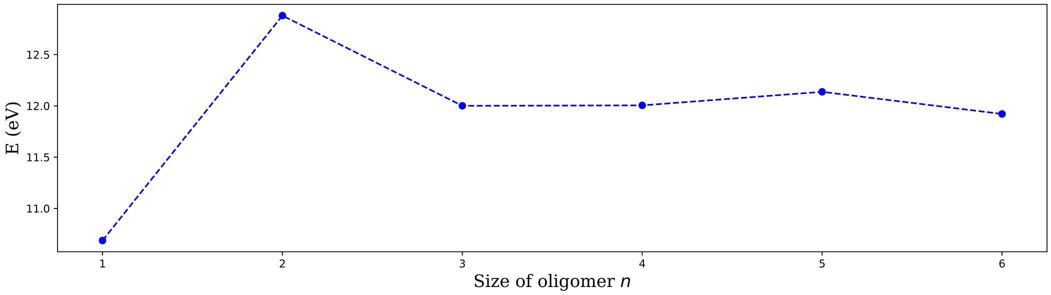

Figure S5: Ionization energies of the neutral $(V_2O_5)_n$ clusters, plotted as a function of oligomer size n.

S6 Geometries of the gas-phase species $(V_2O_5)_n$

This section provides the geometries of all neutral oligomeres up to $n = 6$ as obtained from fully unconstrained DFT optimizations with the ωB97X-V functional. The structures are given in Cartesian coordinates in units of Ångstrom. The formatting supports direct copy and paste from the pdf document.

S6.1 Monomer

```
7
1.out
V -1.98355 0.78849 0.65549
O -0.63894 0.87368 -0.16184
O -2.42118 2.19665 1.20104
O -3.37213 -0.33740 -0.17273
O -4.51815 -1.95738 1.69715
O -2.08721 -0.73773 1.89695
V -3.19712 -1.56570 0.95346
```

S6.2 Dimer

```
14
2.out
V -2.05392 0.72991 0.68844
V 0.27960 -0.59813 -1.01994
O -0.53989 0.77564 -0.24302
O -2.51168 2.10673 1.23949
O -3.33547 -0.19256 -0.13569
O 1.59105 -0.22800 -1.76283
O 0.42230 -2.00806 0.05929
```

```
O -2.37345 -2.97604 0.16796
O -4.50534 -1.97209 1.68623
O -0.40260 -4.30683 -1.31606
O -2.07857 -0.58489 1.88999
O -0.83597 -1.61496 -1.96539
V -3.19342 -1.60212 0.94409
V -0.85990 -2.93015 -0.76427
```

S6.3 Trimer

```
21
3.out
V 0.21497 -0.23427 -1.47505
O -1.02193 0.58878 -0.50763
O 0.52144 0.55729 -2.76928
O 0.42392 -3.81520 0.10489
O -2.25032 -2.87359 0.04351
O -1.24667 -4.42516 -1.97688
O -0.34534 -1.86126 -1.87739
V -0.87146 -3.29519 -0.98804
O 1.65462 -0.34721 -0.44626
V 0.72895 -3.41957 1.80600
V 1.81546 -0.35639 1.31920
V -2.31019 -2.35173 1.73694
V -1.22387 0.71004 1.24956
O 1.34984 -4.62580 2.55100
O 1.81282 -2.02678 1.89687
O -3.57832 -2.89530 2.43822
O 3.11717 0.35590 1.75928
O -2.28954 -0.58584 1.80473
```

```
O -1.81097 2.08656 1.64449
O -0.84247 -2.98598 2.50311
O 0.38859 0.48492 1.95149
```

S6.4 Tetramer

```
28
4.out
V -2.16635 0.99394 0.32600
V 0.39620 -0.12201 -1.46611
O -1.14154 0.66318 -1.07520
O -2.60731 2.47773 0.34987
O -3.58328 -0.06548 0.41300
O 0.96086 0.47799 -2.77510
O 0.04925 -4.22792 0.07977
O -2.05903 -2.38260 0.01033
O -4.76681 -2.51969 0.42461
O -1.51087 -3.99238 -2.14156
O -1.20075 0.57806 1.74114
O 0.13549 -1.86011 -1.67996
V -3.48016 -1.77671 0.85944
V -0.85324 -3.16347 -1.01154
O -3.16047 -2.02450 2.57971
O 1.55033 0.14742 -0.15078
V 0.54688 -3.73532 1.70690
V 1.98069 -0.54610 1.41682
V -1.82331 -2.46824 3.65195
V -0.31362 0.46240 3.26006
O 1.67041 -4.66523 2.22610
O 1.10751 -2.07382 1.52442
```

```
O -2.39126 -3.06750 4.95984
O 3.51098 -0.75236 1.52744
O -0.86488 -1.02890 4.03185
O -0.57741 1.68551 4.17160
O -0.79688 -3.66916 2.85271
O 1.39291 0.41047 2.78712
```

S6.5 Pentamer

```
35
5.out
V 0.85129 -0.44827 -2.07493
O 2.03293 -0.14461 -3.02634
O -0.12971 -3.92028 0.27099
O -0.37366 -4.67934 -2.32893
O 0.66050 -2.19985 -1.92994
V -0.46403 -3.44880 -1.39380
O 1.20114 0.21329 -0.47231
V 0.12084 -3.22620 1.87845
V 0.79307 -0.01503 1.22680
V -2.97571 -2.08120 2.81642
V -2.15678 0.96934 2.56969
O 0.96540 -4.20041 2.73362
O 0.99846 -1.70172 1.70110
O -3.93230 -2.86578 3.74571
O 1.67094 0.89721 2.11796
O -2.75461 -0.44734 3.45176
O -1.56162 1.98880 3.57070
O -1.42900 -2.91750 2.65853
O -0.92028 0.35487 1.46861
```

```
O -2.76101 1.72552 -3.58135
O -4.82826 2.65620 -0.55484
V -2.38484 0.50096 -2.71343
O -0.63781 0.24733 -2.71090
V -4.18510 1.37679 0.03252
O -2.94779 0.72846 -1.05028
O -3.42647 1.71682 1.59416
O -3.23463 -0.92553 -3.31687
O -5.41511 0.11352 0.18047
V -3.52505 -2.44632 -2.45355
V -5.16915 -1.63693 0.25963
O -4.89944 -2.31991 -1.34992
O -2.07662 -2.72620 -1.48308
O -3.70149 -1.88063 1.21391
O -3.74552 -3.58716 -3.47592
O -6.39342 -2.29876 0.93673
```

S6.6 Hexamer

```
42
6.out
V 0.90449 -0.48967 -2.00621
O -2.14110 2.05098 2.86309
O 1.74778 -0.02344 -3.21768
O -0.00077 -4.08431 0.08290
O -3.80106 -2.07983 3.45222
O -0.14084 -4.75020 -2.54796
O 0.77121 -2.25127 -2.01673
V -0.32604 -3.56260 -1.57256
O 1.65801 0.04885 -0.50273
```

```
V 0.28008 -3.34541 1.66200
V 1.51858 -0.27483 1.22768
V -2.05838 -2.11199 3.73552
V -0.82392 0.96046 3.30367
O 0.82945 -4.40187 2.65112
O 1.43440 -2.01697 1.50833
O -1.73179 -3.05117 4.92187
O 2.73785 0.31536 1.97681
O -1.47442 -0.48242 4.08839
O 0.15867 1.67673 4.26163
O -1.22441 -2.64804 2.27267
O 0.01505 0.41632 1.84696
O -1.49837 1.09007 -4.55826
O -4.50756 3.27493 2.32815
O -5.19069 3.03590 -1.29141
V -2.05698 0.52483 -3.22985
V -3.73126 1.95505 2.10137
O -0.73994 0.14834 -2.11353
V -4.35435 1.73686 -1.19546
O -3.15306 1.69444 -2.48905
O -3.54897 1.63158 0.37377
O -2.91221 -0.98943 -3.54082
O -4.64417 0.60400 2.78123
O -5.41989 0.33307 -1.31705
V -3.29012 -2.54631 -2.79631
V -4.96501 -1.11618 2.53830
V -5.58625 -1.33474 -0.75869
O -4.80837 -2.43344 -1.90148
O -1.96681 -2.91649 -1.68554
```

```
O -4.77844 -1.43207 0.80989
O -3.40061 -3.63433 -3.89186
O -6.40507 -1.44777 2.99922
O -7.08644 -1.69009 -0.61996
```

S7 Bulk structures

This section provides the bulk structures as obtained from fully unconstrained DFT optimizations with the PBE functional; see main manuscript for details. We present the undisturbed structure, the structure with one unit removed, and the structure with two units removed. All geometries are given in Cartesian format in units of Ångstrom. Supercell dimensions x, y and z were set to 22.741 Å, 10 Å, and 10.710 Å, respectively. The formatting supports direct copy and paste from the pdf document.

S7.1 Unperturbed plane

```
84

V 0.236201593 2.983430440 2.064976999
V 0.236083242 2.981816470 5.634878377
V 0.236395637 2.981891099 9.204135055
V 2.413247001 1.626961493 0.280567361
V 2.413385231 1.626306315 3.849796577
V 2.413362822 1.625146837 7.419837347
V 5.895491534 1.591168989 0.280257560
V 5.895450629 1.590298672 3.849648302
V 5.895364431 1.589304583 7.419742232
V 8.073364076 2.936400426 2.064915898
V 8.073508938 2.934748366 5.634828035
V 8.073227632 2.935180518 9.204028853
O 9.820918958 2.537922370 2.065758908
O 9.821032425 2.536403058 5.634142654
O 9.820952403 2.537282380 9.203364317
O 4.158773569 2.038586132 0.281133483
```

```
O 4.158851396 2.037884239 3.850428068
O 4.158769084 2.037155127 7.418542400
O 0.354565999 4.558781200 2.065081364
O 0.354330888 4.557178119 5.635952557
O 0.355133510 4.557198784 9.202172527
O 2.296907674 0.051339222 0.282202758
O 2.297007644 0.050716190 3.848523244
O 2.297289182 0.049519705 7.420031125
O 5.986759061 0.013660518 0.281302083
O 5.986471857 0.012703977 3.848150114
O 5.986214044 0.011766184 7.419973758
O 7.946913560 4.511244157 2.065012078
O 7.947020504 4.509620538 5.635656478
O 7.946395056 4.509945293 9.201865020
O 0.548512236 2.434353211 0.279883504
O 0.549102347 2.434103485 3.849858699
O 0.548729288 2.431680043 7.419615881
O 2.098736108 2.177780763 2.065176247
O 2.098259424 2.174969755 5.634860680
O 2.098988837 2.175442666 9.204889591
O 6.216165436 2.131067939 2.065082310
O 6.216576190 2.128345771 5.634798908
O 6.215701970 2.129414727 9.204752635
O 7.765984743 2.381229926 0.279771289
O 7.765570002 2.380563213 3.849835497
O 7.765875028 2.378311436 7.419631857
V 17.236471013 1.896116277 0.279183625
V 13.786334237 1.859726664 0.279326092
O 19.138329001 2.600545632 0.278744756
```

```
O 17.286916353 0.316976199 0.280444597
O 15.505815714 2.372763915 0.279849748
O 13.764721022 0.280015730 0.280527068
O 11.876847087 2.542337871 0.278732165
V 19.489133472 3.119891955 2.063768693
V 11.532336429 3.069907347 2.063623809
O 21.199207119 2.580165660 2.065693239
O 19.488667640 4.699699007 2.063845001
O 17.574515184 2.436820034 2.063903144
O 13.442395362 2.391757846 2.064009172
O 11.541235654 4.649857571 2.063264499
V 17.236240386 1.895691438 3.848548396
V 13.786570385 1.859395311 3.848741988
O 19.137616103 2.600353845 3.848771044
O 17.286226557 0.316520675 3.847234792
O 15.505817471 2.372932639 3.849065297
O 13.765474405 0.279658696 3.847573841
O 11.877481625 2.542293174 3.848793631
V 19.489253019 3.118394553 5.633664308
V 11.532340224 3.068585179 5.633595977
O 21.199179938 2.578414744 5.633738129
O 19.489098922 4.698257133 5.634576794
O 17.574799193 2.434316282 5.633697690
O 13.442204003 2.389499440 5.633813729
O 11.541066190 4.648544845 5.634186358
V 17.236386659 1.894412301 7.418608689
V 13.786522518 1.858156574 7.418786593
O 19.137951215 2.598193050 7.418544725
O 17.286633254 0.315261868 7.418756362
```

```
O 15.505873904 2.371443232 7.417231303
O 13.765113293 0.278434854 7.418970606
O 11.877270667 2.540143861 7.418606652
V 19.489087530 3.118486045 9.202993883
V 11.532585131 3.068694054 9.202905123
O 21.199268839 2.579024302 9.203077071
O 19.488540990 4.698313783 9.201107071
O 17.574136333 2.434901574 9.203671803
O 13.442962710 2.390087921 9.203775207
O 11.541922060 4.648692577 9.200844706
```

S7.2 One unit removed

```
77

V 0.237822914 2.979406333 2.067065634
V 0.254063309 2.983074603 5.637867285
V 0.254280773 2.981287096 9.202296357
V 2.390477013 1.628414302 0.283980673
V 2.380562304 1.622866063 3.851456798
V 2.393454452 1.623761537 7.420496731
V 5.887348998 1.601523206 0.272628445
V 5.873895486 1.605355188 3.864545959
V 5.884939016 1.599488892 7.419862749
V 8.043200286 2.914663647 2.061118389
V 8.116396691 2.885419605 5.659362263
V 8.155933196 2.956732310 9.196339942
O 9.843439449 2.546324954 2.017099683
O 9.905734239 2.486288760 5.762603855
O 9.735375822 2.610902583 9.152611632
```

```
O 4.092179334 2.034039911 0.303114644
O 4.086429316 2.020790136 3.851152447
O 4.092566340 2.028816308 7.398638350
O 0.308593990 4.553046892 2.073496842
O 0.314609244 4.556708822 5.634616763
O 0.316201377 4.555202043 9.196164528
O 2.239623259 0.050758291 0.289469153
O 2.236699045 0.045283745 3.853834986
O 2.241227288 0.046638781 7.414677183
O 5.960566202 0.025635016 0.294982162
O 5.948211849 0.028756726 3.855001897
O 5.957784635 0.023840217 7.405279226
O 7.933183122 4.489847409 2.067123574
O 7.969435212 4.456022727 5.639638252
O 8.026145779 4.560997599 9.193058524
O 0.492892028 2.469600734 0.282923385
O 0.489730220 2.455410663 3.851210066
O 0.501659136 2.454891276 7.420439757
O 2.039788234 2.206878902 2.067232524
O 2.048439735 2.209491668 5.633762479
O 2.049371549 2.198136885 9.206503813
O 6.139011137 2.179383980 2.097314984
O 6.146740963 2.178352705 5.612349930
O 6.106094836 2.073152997 9.203464313
O 7.724911709 2.355847481 0.322647080
O 7.781285950 2.341682088 3.864854958
O 7.716686642 2.340075049 7.378008954
V 17.161773328 1.942691478 0.274483219
V 13.774978077 1.889439813 0.567442645
```

```
O 19.076790057 2.611757532 0.241347034
O 17.280760402 0.365147583 0.280536981
O 15.464339441 2.439270607 0.112517469
O 13.700097020 0.320953289 0.309322423
O 12.195564751 2.585463119 0.164078739
V 19.516703586 3.085055079 2.049886104
V 11.534907303 3.045680956 1.846863761
O 21.164442047 2.500951234 2.061401468
O 19.576694845 4.664412891 2.066188456
O 17.573585215 2.442467881 2.046758374
O 13.599630510 2.404342542 2.245364565
O 11.565659890 4.625138103 1.961174367
V 17.220779126 1.906895080 3.831604909
V 13.771880179 1.888231000 4.099471489
O 19.098155038 2.599733000 3.872839842
O 17.318810714 0.324642534 3.853859659
O 15.538654901 2.390402830 3.903520818
O 13.771758240 0.306933646 3.993288023
O 11.863431458 2.537891651 3.711131192
V 19.552571170 3.096886028 5.598024826
V 11.558265569 3.043917452 5.399755660
O 21.195068139 2.524819331 5.626502061
O 19.588609667 4.676682284 5.636801003
O 17.440738343 2.526951064 5.737637913
O 13.213659467 2.323966686 5.774723096
O 11.635568644 4.617913055 5.613986499
V 17.592148049 1.879230459 7.412496394
O 19.353023874 2.407150478 7.418514686
O 17.355517445 0.319546463 7.409796464
```

```
V 19.552313139 3.093422405 9.233108839
O 21.195713766 2.519261440 9.210590289
O 19.584838076 4.672890864 9.187049804
O 17.435906443 2.522152010 9.091983760
```

S7.3 Two units removed

```
70


V 0.212492288 2.988605214 2.060326026
V 0.225827489 2.989019229 5.652658786
V 0.224714143 2.983281684 9.190162706
V 2.411883683 1.635030560 0.271802155
V 2.352740438 1.619412839 3.854507679
V 2.459342115 1.598559597 7.425372642
V 5.892595301 1.641949175 0.496029386
V 5.835375459 1.621656943 4.025577405
V 8.040075813 2.914478302 1.893122592
V 8.012332165 2.880679322 5.427964738
O 9.828623640 2.538510570 2.060730716
O 9.861168473 2.494630141 5.807055370
O 4.182845843 2.056597500 0.219816843
O 4.122512274 2.027475259 3.851765789
O 4.028403359 1.978034440 7.444323600
O 0.291077928 4.561170212 2.077903526
O 0.299343322 4.560016083 5.633924365
O 0.300695022 4.554291925 9.192026354
O 2.222219183 0.058171758 0.294426573
O 2.220615442 0.036168036 3.857805884
O 2.269799194 0.001220827 7.412462024
```

```
O 5.981790324 0.076368641 0.280237858
O 5.948622783 0.048921274 3.887182195
O 7.921155041 4.485161600 2.034678438
O 7.885036845 4.446344143 5.645965992
O 0.462002646 2.428729484 0.289704994
O 0.460559129 2.420094634 3.836747986
O 0.433483347 2.482770291 7.419767257
O 2.069691520 2.205708297 2.061480150
O 2.048170259 2.226828474 5.586620491
O 2.051238194 2.215118219 9.260042608
O 6.126214988 2.141283170 2.196234016
O 6.377513632 2.180870557 5.717402933
O 7.559169295 2.381169006 0.220668974
O 7.812283867 2.395803881 3.736220554
V 17.200072872 1.918440699 0.293493618
V 13.750469959 1.910372380 0.523607496
O 19.138736353 2.568067202 0.250904540
O 17.341219462 0.345228330 0.292505437
O 15.508200828 2.405284667 0.182863846
O 13.761019049 0.344796299 0.275734622
O 12.094151243 2.527742292 0.194646995
V 19.561880485 3.104897801 2.056745798
V 11.522287478 3.045359782 1.910207136
O 21.204093662 2.505613173 2.067975651
O 19.581454905 4.688220372 2.073947549
O 17.607991507 2.440287273 2.055064802
O 13.544911844 2.402744941 2.201442237
O 11.553472600 4.622388374 2.037050492
V 17.264531281 1.898020664 3.828970304
```

```
V 13.818722214 1.900397347 4.047513542
O 19.144114528 2.562507664 3.865363823
O 17.342243223 0.322095613 3.850861838
O 15.572029969 2.385500372 3.859920563
O 13.799169451 0.325814154 3.892281881
O 11.850629599 2.540426211 3.730126818
V 19.577046573 3.109745959 5.589155834
V 11.534927387 3.014995607 5.439251539
O 21.209825844 2.509536098 5.631379690
O 19.586277263 4.691806997 5.634196280
O 17.483157551 2.475703696 5.687125313
O 13.304773384 2.401764810 5.706123405
O 11.587435039 4.586856851 5.643505725
V 17.550474415 1.899280061 7.418526751
O 19.264529867 2.479981689 7.412917334
O 17.322955030 0.340388863 7.407144516
V 19.576601089 3.104266785 9.244371520
O 21.208213840 2.509844213 9.193832650
O 19.584333871 4.686172608 9.184590476
O 17.480251888 2.466028869 9.143780376
```
